# Malignant Transformation of Non-Neoplastic Barrett's Epithelial Cells through Well-Defined Genetic Manipulations

**DOI:** 10.1371/journal.pone.0013093

**Published:** 2010-09-30

**Authors:** Xi Zhang, Chunhua Yu, Kathleen Wilson, Hui Ying Zhang, Shelby D. Melton, Xiaofang Huo, David H. Wang, Robert M. Genta, Stuart J. Spechler, Rhonda F. Souza

**Affiliations:** 1 Department of Medicine, VA North Texas Health Care System and the University of Texas Southwestern Medical School at Dallas, Dallas, Texas, United States of America; 2 Department of Pathology, VA North Texas Health Care System and the University of Texas Southwestern Medical School at Dallas, Dallas, Texas, United States of America; 3 Caris Life Sciences, Inc., Irving, Texas, United States of America; 4 Harold C. Simmons Comprehensive Cancer Center, University of Texas Southwestern Medical Center at Dallas, Dallas, Texas, United States of America; Technische Universität München, Germany

## Abstract

**Background:**

Human Barrett's cancer cell lines have numerous, poorly-characterized genetic abnormalities and, consequently, those lines have limited utility as models for studying the early molecular events in carcinogenesis. Cell lines with well-defined genetic lesions that recapitulate various stages of neoplastic progression in Barrett's esophagus would be most useful for such studies.

**Methodology/Principal Findings:**

To develop such model cell lines, we started with telomerase-immortalized, non-neoplastic Barrett's epithelial (BAR-T) cells, which are spontaneously deficient in p16, and proceeded to knock down p53 using RNAi, to activate Ras by introducing oncogenic H-Ras^G12V^, or both. BAR-T cells infected with either p53 RNAi or oncogenic H-Ras^G12V^ alone maintained cell-to-cell contact inhibition and did not exhibit anchorage-independent growth in soft agar. In contrast, the combination of p53 RNAi knockdown with expression of oncogenic H-Ras^G12V^ transformed the p16-deficient BAR-T cells, as evidenced by their loss of contact inhibition, by their formation of colonies in soft agar, and by their generation of tumors in immunodeficient mice.

**Conclusions/Significance:**

Through these experiments, we have generated a number of transformed and non-transformed cell lines with well-characterized genetic abnormalities recapitulating various stages of carcinogenesis in Barrett's esophagus. These lines should be useful models for the study of carcinogenesis in Barrett's esophagus, and for testing the efficacy of chemopreventive and chemotherapeutic agents.

## Introduction

The incidence of esophageal adenocarcinoma, a lethal tumor that develops from the metaplastic epithelium of Barrett's esophagus, has increased profoundly in the United States over the past several decades [1], [2]. Knowledge of the molecular events underlying carcinogenesis in Barrett's metaplasia might facilitate the development of effective preventive and treatment strategies for esophageal adenocarcinoma. Unfortunately, the molecular mechanisms underlying the malignant transformation of Barrett's esophagus remain unclear.

Studies on the early molecular events in Barrett's carcinogenesis have been hampered by the lack of appropriate model systems. For example, the results of studies using animal models of Barrett's esophagus, which have involved rats primarily, may not be applicable to humans because of the substantial inter-species differences in esophageal physiology and tissue structure. Human Barrett's cancer cells have sustained numerous, poorly characterized genetic injuries and, therefore, cell lines derived from those malignancies are of limited value for studying early molecular events in carcinogenesis. Biopsy specimens of Barrett's epithelium can be cultured *ex vivo*, but such explants have a short lifespan and cannot be used to study long-term carcinogenetic effects. A durable, *in vitro* model that starts with non-malignant, human, Barrett's metaplastic cells to recapitulate the various stages of neoplastic progression might be most useful for studying the early molecular mechanisms underlying malignancy in Barrett's esophagus.

In 2000, Hanahan and Weinberg characterized six physiologic hallmarks of cancer that normal cells acquire to become malignant [3]. These hallmarks include the ability of cells to provide their own growth signals, to avoid growth inhibitory signals, to resist apoptosis, to replicate without limit, to synthesize new blood vessels, and to invade and metastasize [3]. Recent studies have shown that normal cells can acquire these hallmarks through disruptions in surprisingly few key growth regulatory pathways such as the retinoblastoma (Rb) and p53 pathways, the mitogenic signaling pathways (including Ras), and the telomerase-dependent replicative senescence pathway [4].

We have developed cultures of human Barrett's epithelial cells from esophageal biopsy specimens of non-neoplastic Barrett's metaplasia, and we have immortalized those cells by forcing them to express telomerase. These non-neoplastic cell lines demonstrate spontaneous loss of p16 expression [5]. By introducing additional, well-defined genetic alterations that target the p53 and Ras pathways, we have induced the malignant transformation of our telomerase-immortalized human Barrett's epithelial cells, and we have developed a number of non-transformed cell lines with well-defined, growth-promoting genetic changes that might recapitulate various stages of neoplastic progression in Barrett's esophagus.

## Results

### p53 knockdown alone does not induce malignant transformation of Barrett's epithelial cells

**Figure 1:**
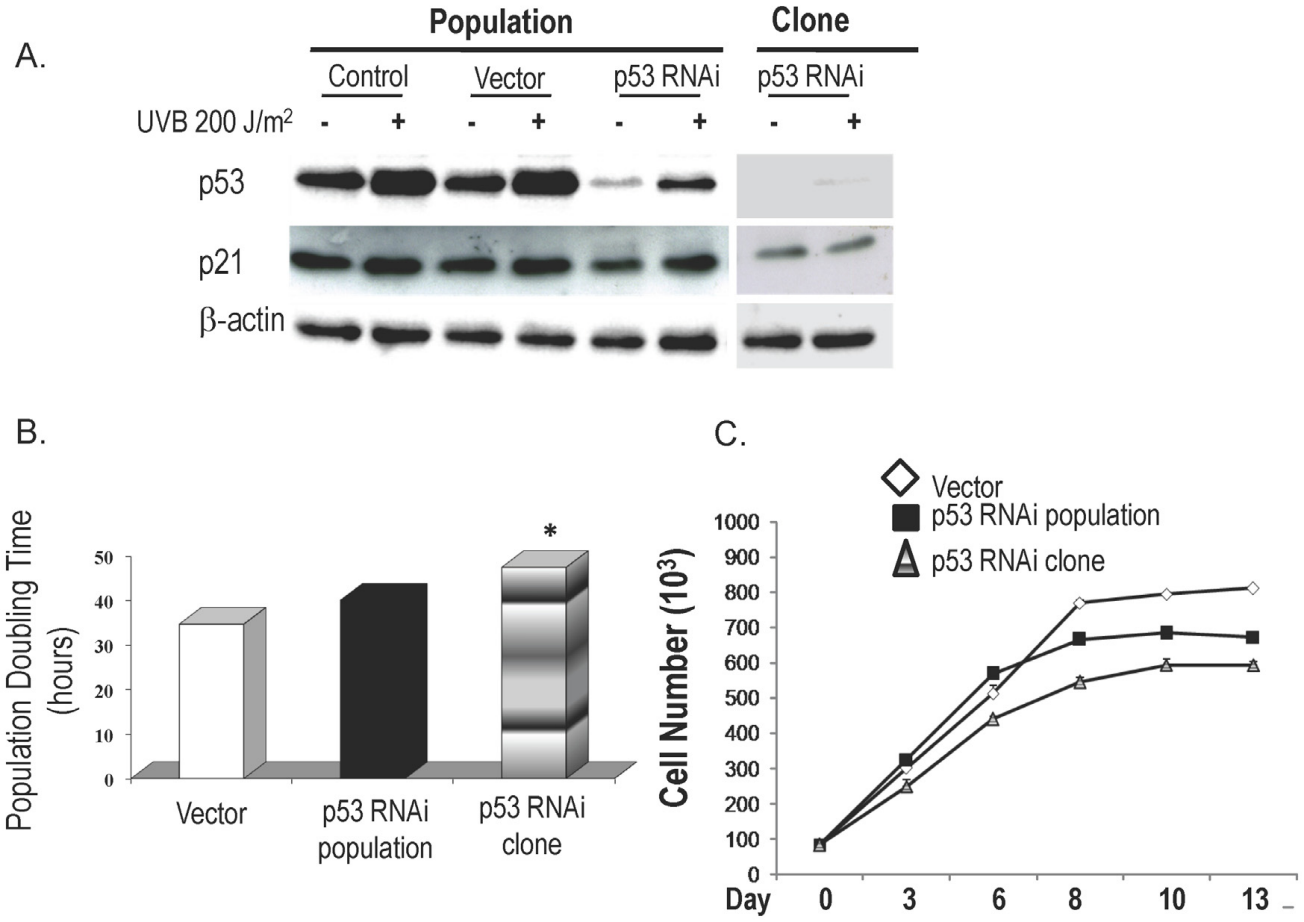
p53 knockdown in BAR-T cells. (A) Western blot demonstrating expression of p53 and p21 at baseline and after irradiation with 200 J/m^2^ UV-B in controls, vector-only cells, the entire p53 RNAi-containing population and a p53 RNAi-containing clone with nearly complete p53 knockdown; β-actin served as a loading control. (B) Population doubling time in BAR-T cells containing p53 RNAi. (*, p<0.05 compared to vector control) (C) Cell-to-cell contact inhibition in BAR-T cells containing p53 RNAi. Cell numbers increase significantly in a time-dependent manner up to day 8 in vector-only cells (p<0.001) and in the p53-knockdown population (p<0.001), and up to day 10 in the clone (p<0.05).

The population of BAR-T cells infected with pSuper-p53RNAi exhibited a marked decrease in baseline p53 expression, with a selected clone showing complete elimination of baseline p53 expression (Figure 1A). In response to UV-B irradiation, a well-known inducer of p53 and p21, there was an increase in p53 and p21 expression in the control and vector-only cells. UV-B evoked a smaller increase in p53 and p21 expression in the p53-knockdown cell population, with the selected clone showing virtually no increase in p53 and p21 expression.

We next determined the population doubling time in BAR-T p53RNAi cells. Normally, functional p53 increases population doubling time and, therefore, we expected our p53 knockdown cells to exhibit decreased doubling times. However, we found no such decrease in doubling time in our p53 knockdown cell population. Indeed, we noted a small, but statistically significant increase in population doubling time in the selected clone (Figure 1B). The explanation for this unanticipated effect is not clear, and further studies are warranted to explore this issue.

Cell to cell contact does not inhibit growth in transformed cells. To seek evidence of malignant transformation in our p53-knockdown BAR-T cells, therefore, we assessed cell to cell contact inhibition. After 8–10 days in culture, vector control and pSuper-p53RNAi knockdown cells (population & clone) all demonstrated a plateau in cell growth characteristic of intact contact inhibition (Figure 1C), suggesting that Barrett's cells with knockdown of p53 are not transformed. As an additional test for *in vitro* transformation, we cultured our p53 knockdown cells in soft agar to see if they exhibited anchorage-independent growth. As a positive control, we used a lung cancer cell line (SEG1), which makes numerous colonies after 3 weeks in soft agar (data not shown). Neither our vector-only nor p53-knockdown BAR-T cells (population & clone) formed colonies in soft agar (data not shown). These findings suggest that p53 knockdown alone does not induce malignant transformation of Barrett's epithelial cells.

### Overexpression of oncogenic H-Ras^G12V^ alone does not induce malignant transformation of Barrett's epithelial cells

**Figure 2:**
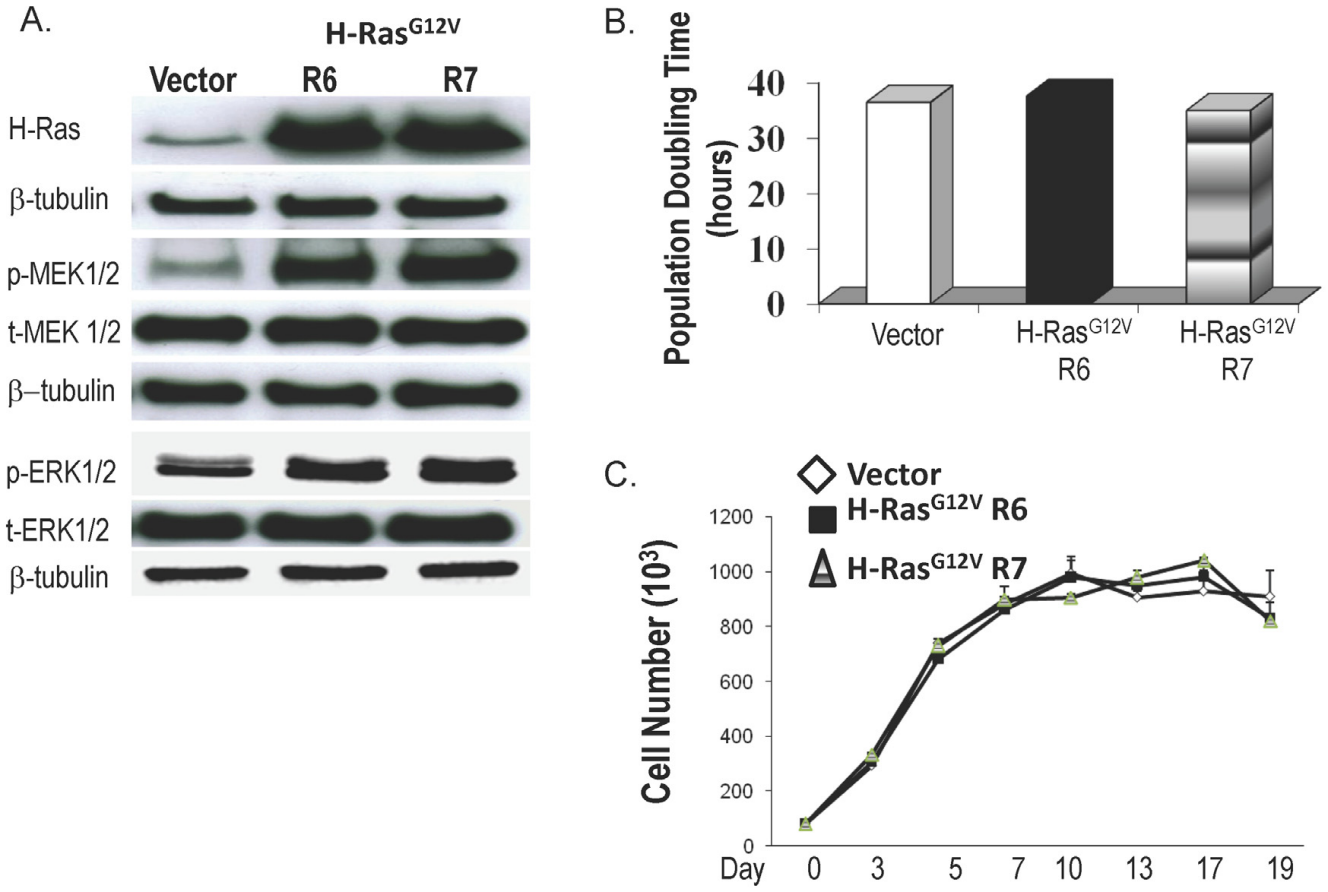
H-Ras^G12V^expression in BAR-T cells. (A) Western blots demonstrating H-Ras expression as well as phosphorylation of two downstream proteins (MEK1/2 and ERK1/2) in a clone containing vector only, and in two H-Ras^G12V^-infected clones(R6 and R7); β-tubulin served as a loading control. (B) Population doubling time in BAR-T cells containing H-Ras^G12V^. (C) Cell–to-cell contact inhibition in BAR-T cells containing H-Ras^G12V^. Cell numbers increased significantly in a time-dependent manner up to day 5 in a clone containing vector only (p<0.05) and up to day 7 in two H-Ras^G12V^-infected clones (R6 and R7; p<0.05).

We infected BAR-T cells with pBabe-H-Ras^G12V^, and determined the expression of H-Ras and the phosphorylation of its downstream proteins (MEK and ERK) in two H-Ras-infected clones (R6 and R7) and in a clone that contained only the vector. We were unable to generate a complete population of oncogenic H-Ras^G12V^-containing cells (despite numerous attempts) because, each time we performed selection for BAR-T infected with pBabe-H-Ras^G12V^, only isolated colonies would survive. As shown in Figure 2A, BAR-T cells infected with H-Ras^G12V^ showed a marked increase in H-Ras expression associated with an increase in phosphorylation of MEK1/2 and ERK1/2, indicating that the H-Ras ^G12V^ was functionally active.

We next determined population doubling time and cell to cell contact inhibition in BAR-T cells expressing H-Ras^G12V^. In full medium containing serum and supplemental growth factors, we found no significant differences in doubling times between our H-Ras^G12V^-expressing clones and the vector control (Figure 2B). As expected, the H-Ras^G12V^-expressing cells showed a growth advantage over vector controls when grown in reduced medium (0.5% of the concentration of serum and growth factors found in full media), further supporting that the H-Ras^G12V^ was functionally active (data not shown). Both our vector control and H-Ras^G12V^-expressing cells demonstrated a plateau in cell growth after 5–7 days in culture, indicating that they maintain contact inhibition (Figure 2C). In addition, the cells did not form colonies in soft agar, demonstrating that oncogenic H-Ras^G12V^ does not induce anchorage-independent growth in BAR-T cells (data not shown). These findings suggest that expression of oncogenic H-Ras^G12V^ alone does not induce malignant transformation of Barrett's epithelial cells.

### p53 knockdown combined with overexpression of oncogenic H-Ras^G12V^ induces malignant transformation of Barrett's epithelial cells

**Figure 3:**
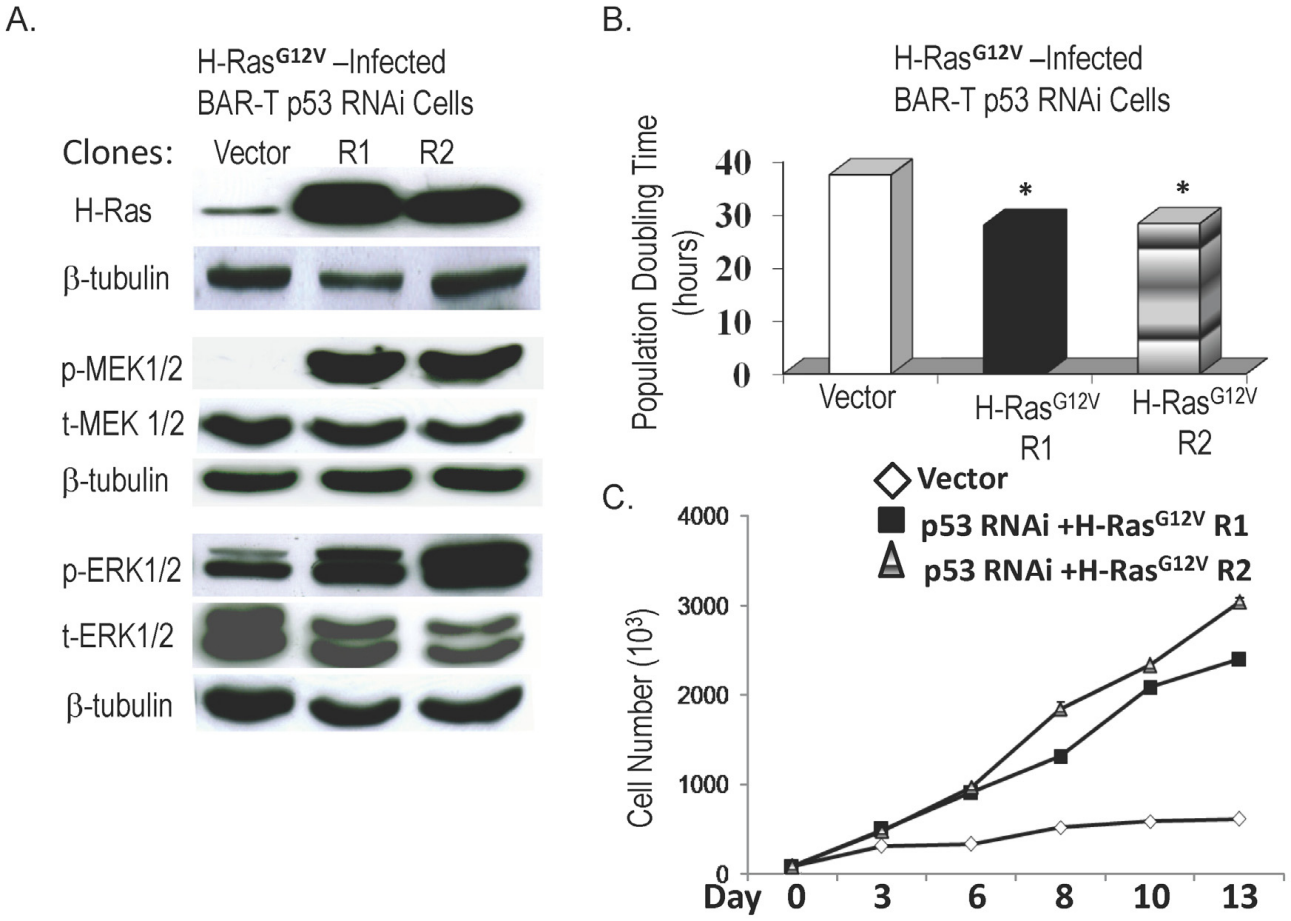
H-Ras^G12V^expression in BAR-T p53 RNAi knockdown cells. (A) Representative western blot demonstrating H-Ras expression as well as phosphorylation of MEK1/2 and ERK1/2 in a clone containing vector only, and in two H-Ras^G12V^-infected, p53 knockdown clones (R1 and R2); β-tubulin served as a loading control. (B) Population doubling time in a clone containing vector only, and in two H-Ras^G12V^-infected, p53 knockdown clones (R1 and R2). (*, p<0.05 compared to vector control) (C) BAR-T p53 RNAi cells expressing H-Ras^G12V^ demonstrate loss of cell-to-cell contact inhibition. In contrast to a clone containing vector only in which cell numbers level off after day 10, cell numbers in the two H-Ras^G12V^-infected, p53 knockdown clones (R1 and R2) continued to increase significantly in a time-dependent manner (p<0.001 for clones R1 and R2).

**Figure 4:**
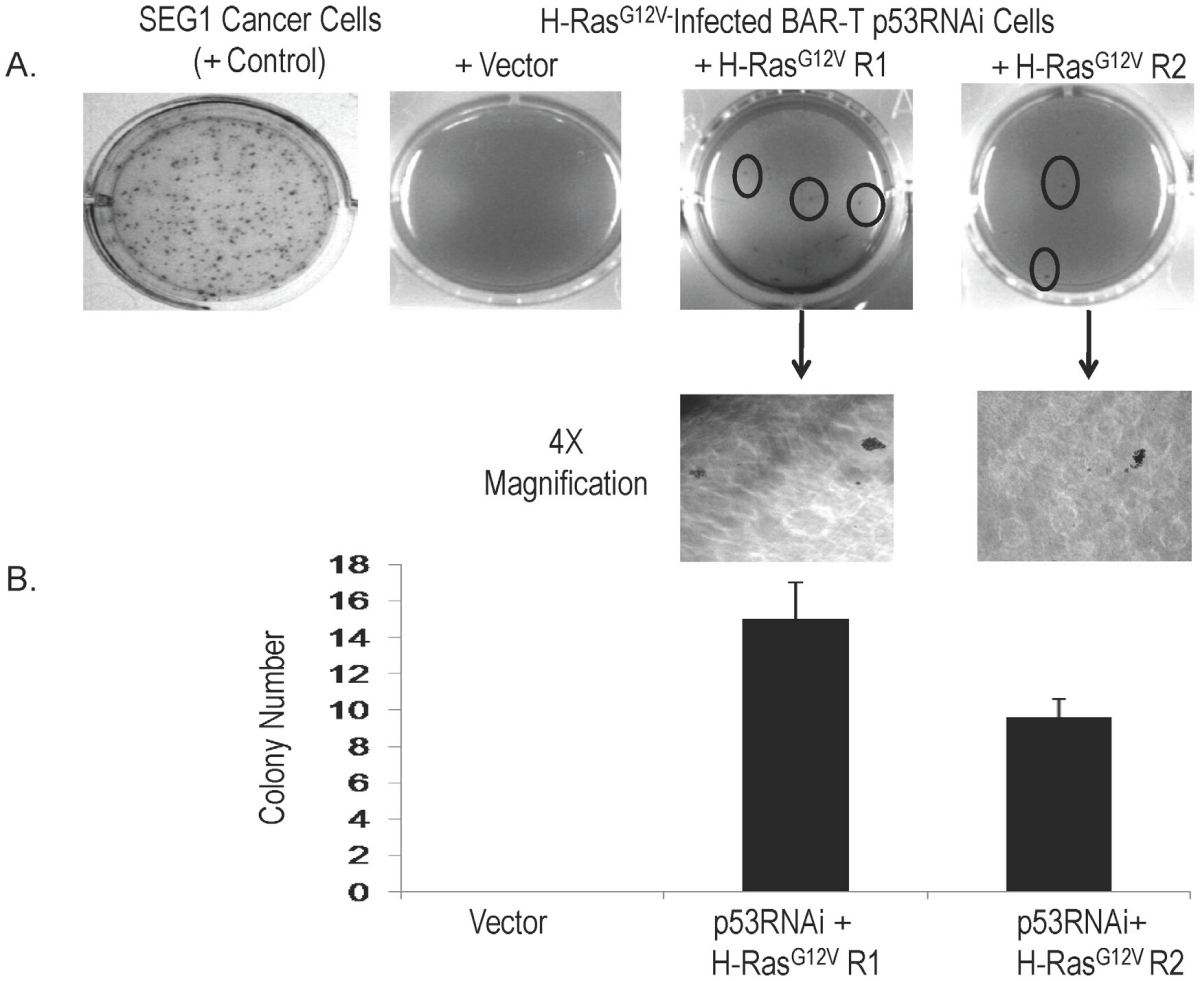
A. Anchorage-independent growth in soft agar of BAR-T p53 RNAi cells expressing H-Ras^G12V^. No colonies are observed for the vector-containing control cells, whereas a number of colonies are observed (circled) for the BAR-T p53 RNAi cells expressing H-Ras^G12V^ (clones R1 and R2). Selected colonies for clones R1 and R2 are shown at higher magnification (4X). B. Quantification of colonies for the vector-containing control cells and for the BAR-T p53 RNAi cells expressing H-Ras^G12V^ (clones R1 and R2). The SEG1 cancer cells formed 325.3±14.4 SEM colonies and served as a positive control.

**Figure 5:**
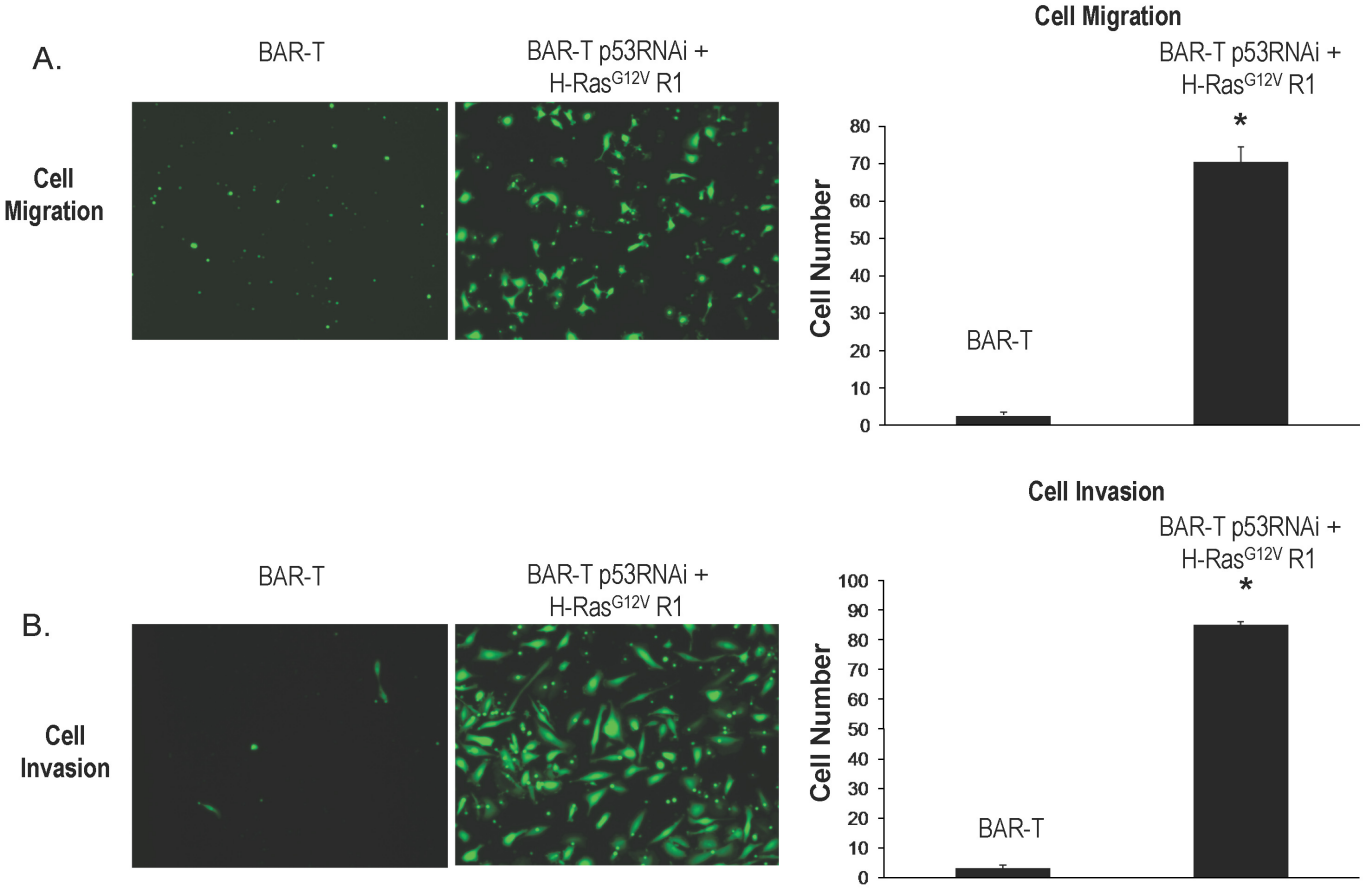
A. Migration of BAR-T p53 RNAi cells expressing H-Ras^G12V^ clone R1 cells. Visualization by the fluorescent dye calcein AM of cells that have migrated through the membrane; quantification of cell migration for BAR-T p53 RNAi cells expressing H-Ras^G12V^ clones R1 and BAR-T control cells. (B) Invasion of BAR-T p53 RNAi cells expressing H-Ras^G12V^ clone R1 cells. Visualization by the fluorescent dye calcein AM of the cells that have invaded through the membrane; quantification of cell invasion for BAR-T p53 RNAi cells expressing H-Ras^G12V^ clones R1 and BAR-T control cells. Bar graphs depict the mean + SEM. (*, p<0.0001 compared to control).

Having found that neither p53 knockdown alone nor expression of oncogenic H-Ras^G12V^ alone induced malignant transformation of our BAR-T cells, we next combined these genetic alterations. We used our BAR-T p53RNAi-expressing clone for infection with oncogenic H-Ras^G12V^. We then selected one vector-only containing clone and two additional H-Ras^G12V^-expressing clones (R1 and R2) for further characterization. There was a marked increased in expression of H-Ras, phospho-MEK1/2 and phospho-ERK1/2 in BAR-T p53RNAi cells infected with H-Ras^G12V^ (Figure 3A). The clones with combined H-Ras^G12V^ expression and p53 knockdown exhibited a significant decrease in population doubling time (Figure 3B), and a loss of cell to cell contact inhibition (Figure 3C). After 3 weeks of culture in soft agar, we observed several colonies of our BAR-T p53RNAi H-Ras^G12V^-expressing clones, whereas the vector control cells did not form colonies (Figure 4). To determine whether the BAR-T p53RNAi H-Ras^G12V^-expressing clones have the cancer cell abilities of migration and invasion, we used the BAR-T p53RNAi H-Ras^G12V^ clone R1 cells for migration and invasion assays. Compared to BAR-T control cells, transformed BAR-T p53RNAi H-Ras^G12V^R1 cells demonstrated a significant increase in cell migration and invasion (Figure 5 A and B). These data suggest that oncogenic H-Ras^G12V^ in combination with p53 knockdown induces neoplastic transformation of Barrett's epithelial cells.

**Figure 6:**
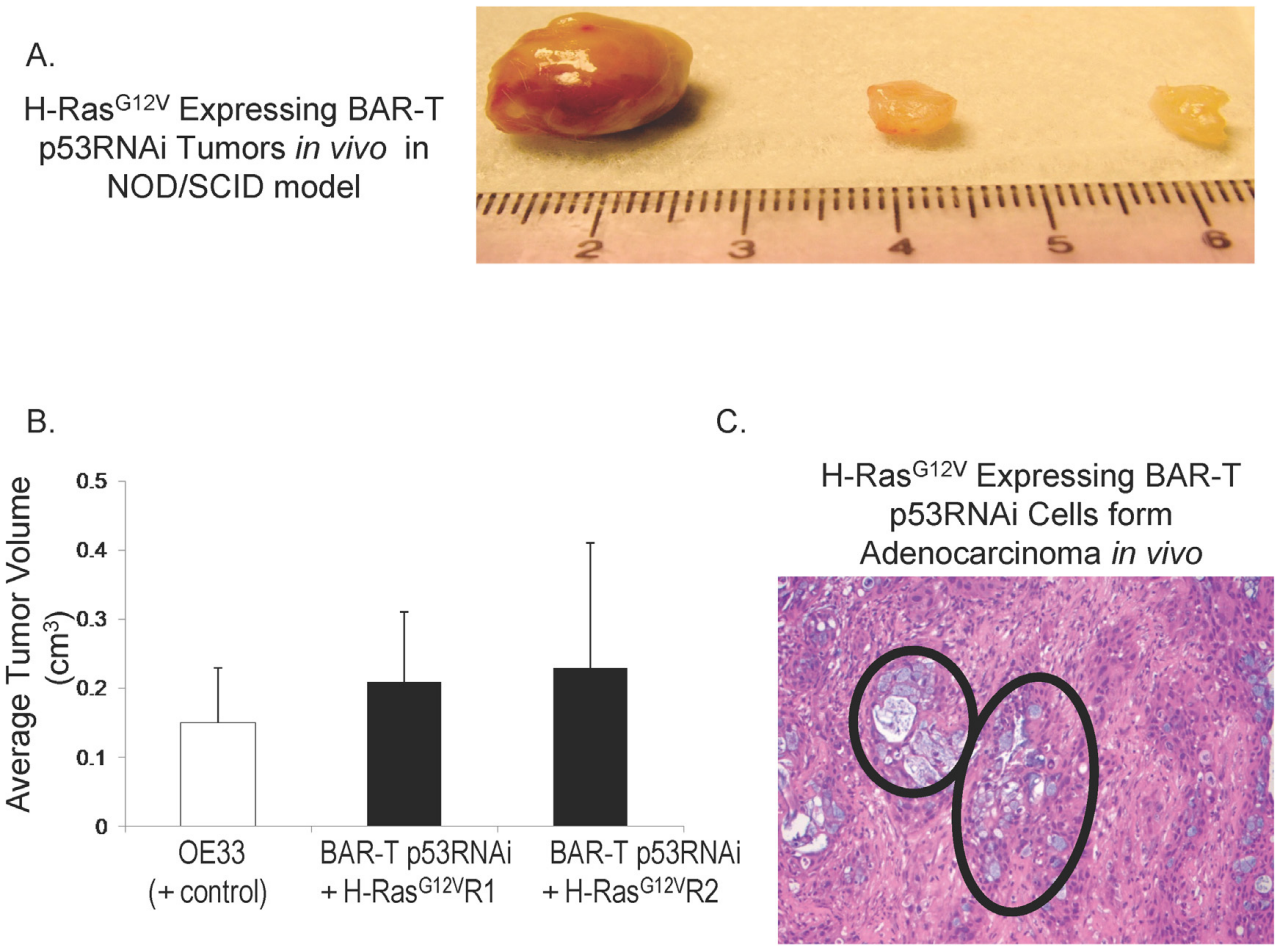
*In vivo* tumor formation by BAR-T p53 RNAi cells expressing H-Ras^G12V^. (A) Three representative tumors formed by BAR-T p53 RNAi cells expressing H-Ras^G12V^ clone R1 in the NOD/SCID mouse model. (B) There were no significant differences between the average volumes of the *in vivo* tumors formed by the OE33 cancer cells and those formed by BAR-T p53 RNAi cells expressing H-Ras^G12V^ clones R1 and R2 (p = .88). (C) Histology of the tumors demonstrating mucin-containing glands (circled) typical of an adenocarcinoma.

**Figure 7:**
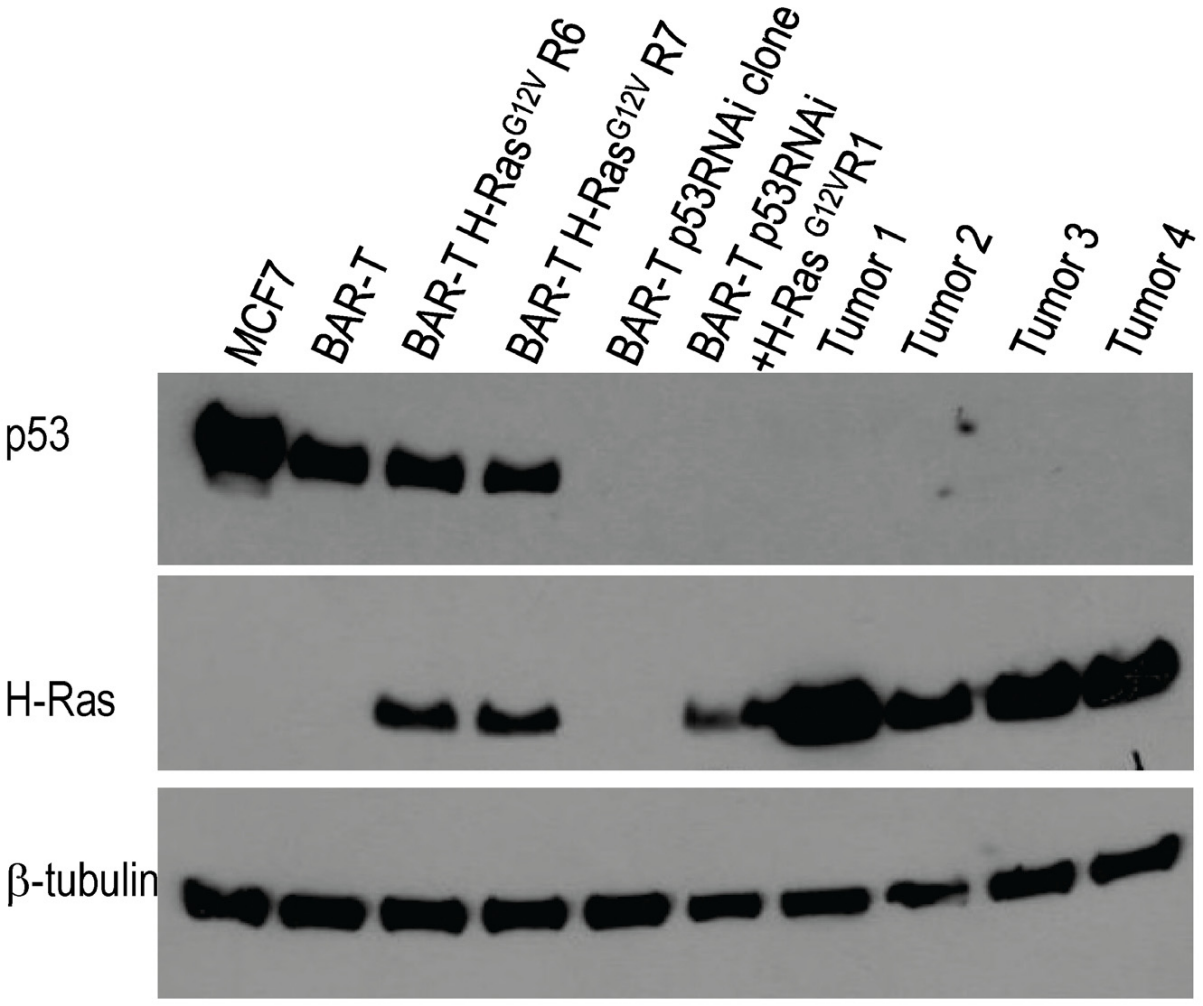
Western blots demonstrating expression of p53 and H-Ras in BAR-T cells, two H-Ras^G12V^-infected clones (R6 and R7), p53 RNAi-containing clone, BAR-T p53 RNAi cells expressing H-Ras^G12V^ (clone R1), and tissue from 4 xenograft tumors (Tumor 1–4) derived from BAR-T p53 RNAi cells expressing H-Ras^G12V^ (clone R1). Note that tissue samples from all 4 xenograft tumors demonstrate knockdown of p53 and expression of H-Ras. β-tubulin served as a loading control; MCF7 cells served as a positive control for p53 expression.

The “gold standard” assay to define tumorigenicity is the ability of cells to form tumors in immunodeficient mice. Therefore, we injected BAR-T p53RNAi H-Ras^G12V^-expressing clones (R1 and R2) subcutaneously into Nu/Nu mice (T cell deficient) and NOD/SCID mice (T, B, and NK cell deficient as well as complement deficient). SEG-1 cells served as a control for the Nu/Nu mice; OE33 cells served as a control for the NOD/SCID mice. Unlike the vector-containing control cells (BAR-T p53RNAi cells containing the empty vector p-Babe-zeocin), the cells with p53 knockdown and H-Ras^G12V^ expression formed tumors in both the Nu/Nu (Table 1) and the NOD/SCID mice within 10–14 weeks (Table 1 and Figure 6A). There were no significant differences between the average volumes of the *in vivo* tumors formed by the OE33 cancer cells and those formed by BAR-T p53 RNAi cells expressing H-Ras^G12V^ clones R1 and R2 (Figure 6B). Histological evaluation of the tumors revealed mucin-containing glands typical of an adenocarcinoma (Figure 6C). To confirm that the xenograft tumors maintained p53 knockdown and overexpression of oncogenic H-Ras^G12V^, Western blots for p53 and H-Ras were performed on tissue from 4 individual xenograft tumors derived from the BAR-T p53RNAi+H-Ras^G12V^R1 cells. Similar to the BAR-T p53RNAi+H-Ras^G12V^ R1 cells *in vitro*, the xenograft tumor tissues derived from these cells lacked p53 expression and had elevated levels of H-Ras expression (Figure 7). These data demonstrate that the molecular changes introduced into the BAR-T cells *in vitro* (p53 knockdown and overexpression of oncogenic H-Ras^G12V^) were maintained by the cells that generated the tumors *in vivo*.

**Table 1:** H-Ras^G12V^-Expressing BAR-T p53RNAi Cells Are Tumorigenic In Vivo.

Cell Line	Tumors/Injections NOD/SCID Mice (↓T, B, NK cells, ↓complement)	Tumors/Injections Nude Mice (↓T cells)
BAR-T p53 RNAi +Vector	0/4 (26 weeks)	0/4 (26 weeks)
BAR-T p53 RNAi + HRasG^12V^R1	4/4 (10 weeks)	3/4 (10 weeks)
BAR-T p53 RNAi + HRasG^12V^R2	3/4 (12 weeks)	3/4 (14 weeks)
BAR10-T p53 RNAi + HRasG^12V^R5	6/6 (6 weeks)	3/4 (6 weeks)

To confirm that p53 knockdown and overexpression of oncogenic H-Ras^G12V^ induce malignant transformation of Barrett's epithelial cells, we infected a second telomerase-immortalized Barrett's epithelial cell line (BAR10-T) with pSuper-p53RNAi followed by infection of the population of p53RNAi-expressing cells (rather than a selected clone) with pBabe- H-Ras^G12V^. Following selection with zeocin, we selected a resultant p53RNAi H-Ras^G12V^-expressing clone (R5) for characterization of *in vivo* tumorigenesis. These cells produced tumors in both the Nu/Nu and the NOD/SCID mice within 6 weeks (Table 1). Histological analysis showed that the tumors consisted of moderately to poorly differentiated carcinoma with areas of both squamous and glandular differentiation. Portions of the tumor also exhibited goblet cells, poorly differentiated sarcomatoid features, and necrosis (data not shown).

### Transformed Barrett's cells have clonal chromosomal abnormalities

**Figure 8:**
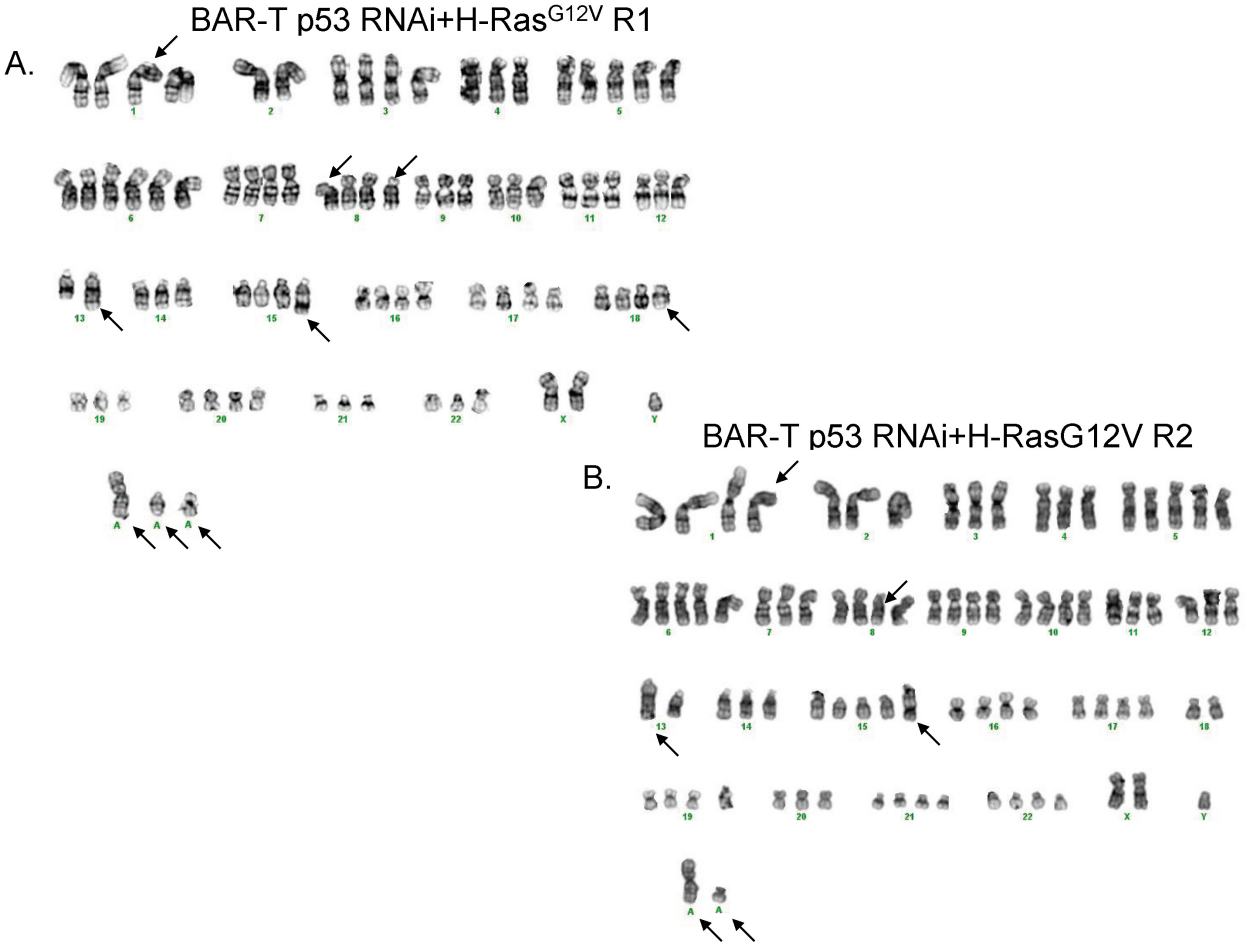
Representative karyotype of BAR-T p53 RNAi + H-Ras^G12V^ cells prior to their use in the *in vivo* tumorigenesis assays (A) clone R1 and (B) clone R2. Structural aberrations are designated with an arrow.

Conventional cytogenetic analysis identified complex clonal chromosome aberrations in all three transformed cell lines. Our analysis of 20 metaphase cells for each tumor revealed for the BAR-T p53RNAi H-Ras^G12V^-expressing clone R1 a 46∼53,XY,+X,add(1)(p12),+5,+6,+9,−14,+15,add(15)(q24),+19,+20,+21,+1∼3mar[cp3]/61∼80,XY,-X,+3,+5,+6,+7,+8,add(8)(p11.2),del(8)(p11.2p23),+9,del(12)(q15q24.3),add(13)(q32), i(13)(q10), −14,+19,+20,+1∼4mar[cp3]/83∼89,XXY,-Y,add(1)(p12),del(1)(q12), −2,del(2)(q11.2), −4,+5,+6,der(7)t(7;13)(q11.2;q12),add(8)(p11.2),del(8)(p11.2p23),+9, −11, −12,del(12)(q15q24.3), −13,add(13)(q32),i(13)(q10), −14,der(14)t(14;22)(p11.2;q11.2), −15,add(15)(q24), −16, −18, add(18)(q11.2), −19,+20, −21,add(21)(q22), −22,+2∼7mar[14] karyotype (Figure 8A); for the BAR-T p53RNAi H-Ras^G12V^-expressing clone R2 a 55∼79<2n>,XY,+X,add(1)(p12),+2,+3,+4,+5,+5,+5,+6,+6,+7,del(8)(p11.2p23),+9,+9,+10,+10,+11,+12,add(13)(q32),i(13)(q10),der(14)t(14;22)(p11.2;q11.2),add(15)(q24),+16,+17,+19,+20,+20,+21, −22[cp7]/81∼89<4n>,XXY,-Y, −1,add(1)(p12), −2, −4,+5,+6, −7,del(8)(p11.2p23),+9, −10, −11, −12,add(13)(q32),i(13)(q10),add(15)(q24), −16, −17, −18, −18,+20, −21, −22, −22[cp13] karyotype (Figure 8B); and for the BAR-10T p53RNAi H-Ras^G12V^-expressing cells a 91,XXYY<4n>,−3,+5,+8, −9, −13, −14, −21,+2mar[20] karyotype. Thus the Barrett's cells transformed *in vitro* displayed clonal chromosomal abnormalities consistent with neoplasia.

## Discussion

We have shown that malignant transformation of benign Barrett's epithelial cells can be achieved *in vitro* through disruption of relatively few key growth regulatory pathways including the p16/Rb and p53 checkpoint arrest pathways, the mitogenic Ras signaling pathway, and the telomerase-dependent replicative senescence pathway. As a result of these experiments, we have established a series of transformed and non-transformed Barrett's epithelial cell lines that can be used to elucidate the contribution of specific genetic alterations, alone and in combination, to carcinogenesis in Barrett's esophagus. Moreover, our series of Barrett's epithelial cell lines with well-defined genetic alterations can be used to explore the molecular mechanisms by which environmental factors (e.g. acid and bile exposure) promote cancer development in Barrett's esophagus, and to test the efficacy of chemopreventive and chemotherapeutic agents.

The molecular alterations that have been proposed to contribute to carcinogenesis in Barrett's esophagus can be patchy in distribution and heterogeneous in nature [6]. Consequently, the identification of those molecular abnormalities can be heavily influenced by biopsy sampling error. This problem confounds attempts to determine the precise order in which genetic alterations accumulate. Nevertheless, available studies using biopsy specimens from patients with Barrett's esophagus support a carcinogenetic role for alterations in the same pathways that caused transformation of our non-neoplastic Barrett's epithelial cells *in vitro*, i.e. the p16/Rb and p53 checkpoint arrest pathways, the mitogenic Ras signaling pathway, and the telomerase-dependent replicative senescence pathway.

Cellular immortalization has been proposed as an early step in the neoplastic transformation of human cells [7]. A number of normal cell types (including esophageal squamous cells) have been immortalized through the forced expression of telomerase alone or in combination with the insertion of viral oncoproteins such as the SV40-early region [8], [9]. Viral oncoproteins can cause a substantial number of poorly characterized genetic alterations [10], however, a feature that limits the utility of those cells for studies on the contribution of specific genetic alterations. Cells immortalized with human telomerase have shown no signs of tumorigenesis, altered differentiation, or deregulation of cell proliferation [11]. Moreover, expression of telomerase RNA has been detected in tissue samples of non-dysplastic, Barrett's metaplasia suggesting that telomerase activation is an early event in Barrett's carcinogenesis [12], [13]. Thus, we selected to bypass the replicative senescence pathway by immortalizing our Barrett's epithelial cells (BAR-T and BAR10-T) solely through the introduction of human telomerase (hTERT).

Functional disruption of the Rb pathway can be achieved by inactivation of p16, which is the earliest and most common genetic alteration described in patients with Barrett's esophagus [14]. Biopsy specimens of non-neoplastic Barrett's metaplasia demonstrate inactivation of p16 via promoter methylation, loss of heterozygosity (LOH), or mutation in 73% to 87% of patients [15], [16]. Therefore, it is not surprising that both our BAR-T and BAR10-T cell lines exhibit spontaneous loss of p16 expression. The “ideal” *in vitro* model of Barrett's metaplasia in which to study the effects of well-defined genetic alterations on neoplastic transformation would be one in which p16 expression remains intact, with the deficiency in p16 expression being genetically engineered, rather than spontaneously acquired. Spontaneous loss of p16 expression does not negate our attempts at establishing an *in vitr*o model of neoplastic progression in Barrett's esophagus however, as p16 inactivation is an early genetic event in metaplastic Barrett's epithelial cells *in vivo* [14], [16]. Our BAR-T and BAR10-T cell lines also express Barrett's epithelial cell differentiation markers such as villin and cytokeratins 4, 8, and 18, and both lines exhibit contact inhibition and anchorage-dependent growth. Thus, our telomerase-immortalized, p16-deficient BAR-T and BAR10-T cell lines are not transformed and appear to be a good model for non-neoplastic Barrett's metaplasia.

In an attempt to transform our non-neoplastic Barrett's epithelial cells, we knocked down the p53 pathway using a specific p53RNAi expression vector. This was done to recapitulate the p53 allelic loss that appears to occur frequently during carcinogenesis in Barrett's esophagus. In biopsy specimens of non-dysplastic Barrett's metaplasia, allelic loss of p53 occurs more frequently than p53 mutation and, in the absence of p53 allelic loss, p53 mutations are rare [16], [17]. Loss of p53 is found frequently in biopsy specimens of Barrett's esophagus that also exhibit p16 inactivation, and the limited data available on the order in which these genetic alterations accumulate suggest that loss of p53 follows the inactivation of p16 [16]. In our BAR-T cells, which are already p16 deficient, specific knockdown of the p53 pathway alone did not induce features of neoplastic transformation. This finding is consistent with reports on other human epithelial cells (including esophageal squamous, embryonic kidney, and mammary cells) in which the introduction of telomerase in combination with the SV40 early region (a viral oncoprotein that knocks down the Rb and p53 pathways) results in immortalized, but not transformed cells [9], [10], [18].

We also activated the mitogenic Ras signaling pathway in our non-neoplastic Barrett's cells by introducing an expression vector containing oncogenic H-Ras^G12V^. Involvement of the Ras pathway in the early stages of neoplastic progression in Barrett's esophagus has been suggested by studies demonstrating genomic amplification or overexpression of the epidermal growth factor receptor (EGFR) and its ligand, transforming growth factor alpha (TGF-α), in biopsy samples of non-dysplastic Barrett's metaplasia [19], [20]. Although specific K-Ras mutations and expression of oncogenic H-Ras are rarely detected in non-neoplastic Barrett's epithelium, both of these abnormalities are found frequently in dysplastic Barrett's epithelium and in esophageal adenocarcinomas [21]–[25].

In some primary epithelial cells, activation of oncogenic Ras causes oncogene-induced senescence, a form of growth arrest that appears to prevent cancer formation [26]. In telomerase-immortalized esophageal squamous epithelial cells, H-Ras^G12V^-induced senescence has been shown to be mediated via the upregulation of p16 [27]. Thus, it is not surprising that our BAR-T cells, which are p16 deficient, did not exhibit growth arrest after the introduction of H-Ras^G12V^. Indeed, population doubling times for those cells did not differ significantly from those of vector-containing control cells. The oncogenic H-Ras^G12V^ produced by our Barrett's cells was functional, as evidenced by an associated increase in the levels of phosphorylation of the downstream proteins MEK1/2 and ERK1/2. Nevertheless, oncogenic Ras expression alone did not transform our BAR-T cells. Similar findings have been reported for a telomerase-immortalized human fibroblast cell line in which the combination of p16 downregulation and expression of oncogenic H-Ras^G12V^ did not induce neoplastic transformation [28].

A number of primary human epithelial cells, including esophageal squamous cells, have been transformed using a combination of viral oncoproteins, hTERT, and oncogenic Ras [9], [18], [29], [30]. Few reports have documented the transformation of human epithelial cells in the absence of viral oncoproteins [31]. Without using viral oncoproteins, we have achieved transformation of telomerase-immortalized, non-neoplastic, human Barrett's epithelial cells, which are deficient in p16, by introducing a combination of p53 knockdown and oncogenic H-Ras^G12V^ expression. When the transformed cells were injected into immunodeficient mice, the resulting tumors demonstrate histological phenotypes similar to those of Barrett's-associated esophageal adenocarcinomas. We also found that the transformed Barrett's cells had clonal chromosomal abnormalities consistent with malignancy. It is possible that the chromosomal abnormalities associated with the knockdown of p53 and the insertion of oncogenic H-Ras^G12V^ contributed to malignant transformation.

In conclusion, without using viral oncoproteins, we have induced the malignant transformation of human hTERT-immortalized Barrett's epithelial cells, which are deficient in p16, through the knockdown of p53 and the forced expression of oncogenic H-Ras ^G12V^. Similar genetic alterations are found frequently in esophageal biopsy specimens from patients with various stages of neoplasia in Barrett's esophagus. Through these experiments, we have generated a number of transformed and non-transformed human Barrett's epithelial cell lines with well-characterized genetic abnormalities that can be used as models for the study of carcinogenesis in Barrett's esophagus, and for testing the efficacy of chemopreventive and chemotherapeutic agents.

## Materials and Methods

### Ethics Statement

Experimental methods using mice were approved by the Institutional Animal Care and Use Committee at the Dallas VA Medical Center under ACORP #05-049.

### Cell culture

We used 2 non-neoplastic, telomerase-immortalized Barrett's epithelial cell lines (BAR-T and BAR10-T) that were established in our laboratory from endoscopic biopsy specimens of non-dysplastic Barrett's specialized intestinal metaplasia taken from two patients with long-segment Barrett's esophagus [5], [32]. Like BAR-T cells [5], our BAR10-T cells express Barrett's epithelial cell differentiation markers such as villin and cytokeratins 4, 8, and 18, develop spontaneous loss of 16 protein expression, demonstrate cell to cell contact inhibition, and do not exhibit anchorage-independent growth in soft agar (data not shown). Conventional cytogenetic analysis identified a 47, XY, +5[7]/90–93<4n>, XXYY,-8[cp5]/46,XY[18] karyotype in BAR10-T cells. BAR cell lines were co-cultured with a fibroblast feeder layer and maintained in supplemented keratinocyte basal media, KBM2, (Lonza, Walkersville, MD) as previously described [5], [33]. Cells were equally seeded into collagen IV-coated wells (BD Biosciences, San Jose, CA) for individual experiments. Unless otherwise specified, we used the BAR-T line for most experiments because of the extensive characterization of this line done by our laboratory [5], [32], [34]–[36].

### Viral vectors and vector transduction

For knockdown of p53, we generated pSUPER-RNAi-p53 by cloning the DNA fragment containing the p53 RNAi sequence from the pSUPER.p53 plasmid (OligoEngine, Seattle, WA) into the EcoR1/HindIII cloning site of the pSUPER.retro.neo retroviral mammalian expression vector (OligoEngine) as previously described [34]. The pBabe-puromycin-based retroviral vector expressing human oncogenic H-Ras^G12V^ and pBabe-zeocin were obtained from Dr. Robert Weinberg (Whitehead Institute, Cambridge, MA)[30]. The DNA fragment containing oncogenic H-Ras^G12V^ was digested (EcoR1/BamH1, Roche, Indianapolis, IN) from pBabe-puromycin and cloned into the EcoR1/BamH1 cloning site of the pBabe-zeocin retroviral mammalian expression vector generating the retroviral vector pBabe- H-Ras^G12V^ –zeocin; pBabe-zeocin without the insert served as a control. pBabe- H-Ras^G12V^ –zeocin was transformed into competent bacterial cells (Subcloning Efficiencey DH5α, Invitrogen) per the manufacturer's instructions. Ampicillin-resistant colonies were selected, plasmid was isolated, and the presence of the insert was confirmed by EcoRI and BamH1 digestion and DNA sequencing. Retroviral particles were generated as previously described [8]. BAR-T and BAR10-T cells were infected at approximately 50% confluence in the presence of 4 µg/ml of Polybrene (Sigma, St. Louis, MO) for 10–12 hours. After recovery for 72 hours, cells were selected in 60 µg/ml G418 or 80 µg/ml zeocin for 10 days. Cell clones were selected using cloning cylinders. We generated BAR-T cells containing 1) pSUPER-retro.neo (vector control); 2) pSUPER-p53RNAi; 3) pBabe-zeocin (vector control); 4) pBabe- H-Ras^G12V^ –zeocin; 5) pSUPER-p53RNAi and pBabe-zeocin; and 6) pSUPER-p53RNAi and pBabe- H-Ras^G12V^ –zeocin.

### UV-B irradiation

UV-B irradiation was performed as previously described [34]. Cells were irradiated with 200 J/m^2^ of UV-B and 24 hours later, cells were collected for Western blot analysis.

### Western blotting

Cells were lysed in 1X cell lysis buffer (Cell Signaling Technology, Beverly, MA); protein concentrations were determined using the BCA-200 Protein Assay kit (Pierce, Rockford, IL). Equal amounts of protein were separated by SDS-polyacrylamide gel electrophoresis and transferred to nitrocellulose membranes. The membranes were incubated with primary antibodies (1∶1,000 dilutions) to p53, H-Ras, phospho- and total MEK1/2, and phospho- and total ERK1/2 or (1∶500 dilutions) p21 (Cell Signaling Technology). Horseradish peroxidase secondary antibodies were used and chemiluminescence was determined using the Super Signal West Dura detection system (Pierce, Rockford, IL); β-actin or β-tubulin (Sigma) was used to confirm equal loading. All Western blots were performed in duplicate.

### Growth rate and population doubling time

Cell numbers were determined using a Z1 particle counter (Beckman Coulter, Fullerton, CA). Population doublings and doubling times were determined using the formulas PD = log (N_t_/N_0_)/log (2) and DT (hours)  =  (t–t_0_)/PD where t_0_ = time at which the cells were seeded, t = time in hours, N_t_ = cell number at time t, and N_0_ = initial cell number seeded, respectively. All experiments were performed in duplicate.

### Cell to cell contact inhibition

Cell to cell contact inhibition was performed as previously described [5]. In brief, 83×10^3^ cells per well of a 6 well plate were seeded and placed in the incubator for at least10 days. Cell counts were performed using a Z1 particle counter at various time points. All experiments were performed in duplicate.

### Soft agar assay

The soft agar assay was performed as previously described [5]. In brief, 1000 cells were added to 2.5 ml of Noble agar (Sigma) which had been kept at 45°C (final concentration of 0.33% (w/v) agar) and supplemented with 20% serum. The cell-agar mixture was plated in duplicate onto dishes containing a solidified.5 ml layer of 0.5% agar-cell culture medium mix. Cells were fed weekly with growth media and plates were examined daily for 3 weeks. Plates were imaged with a Bio-Rad Molecular Imager (Bio-Rad, Hercules, CA, USA). SEG-1 lung adenocarcinoma cells were used as a positive control. All experiments were performed in duplicate.

### Migration and invasion Assays

For both migration and invasion assays, cells were equally seeded onto a BD Falcon FluoroBlok 24-Multiwell Insert plate with an 8.0 µ pore size; for the invasion assay the plate was coated with BD Matrigel Matrix (BD Biosciences, Bedford, MA). For both assays, KBM-2 growth media was placed in the bottom of the wells as a chemoattractant. After 3 hours, the cells on the bottom of the filters were labeled with the fluorescent dye calcein AM (4 µg/ml). Images were obtained from 2 separate high power fields from three individual wells and cells were counted to determine the proportion which migrated or invaded through the membrane.

### Cytogenetic analysis

Cytogenetic analysis was performed on BAR-T p53 RNAi H-Ras^G12V^-expressing clones R1 and R2, and BAR-10T p53 RNAi H-Ras^G12V^-expressing clone R5 cells prior to their use in the *in vivo* tumorigenesis assays. Dividing cells were harvested from cultures incubated without mitogen, and Trypsin G-banded using standard methods [37]. Briefly, metaphase BAR-T p53 RNAi H-Ras^G12V^-expressing clones R1 and R2, and BAR-10T p53 RNAi H-Ras^G12V^-expressing clone R5 cells were obtained by colcemid arrest followed by hypotonic treatment with pre-warmed 0.075M KCl. They were then fixed and washed in freshly made modified Carnoy's fixative (3∶1 absolute methanol:glacial acetic acid), dropped onto pre-cleaned, wet microscope slides and air-dried. Cytogenetic abnormalities were classified according to the International System for Human Cytogenetic Nomenclature [38].

### In vivo tumorigenesis

Female 6 week old, specific pathogen free, nude mice (nu/nu) and NOD/SCID mice were obtained (Charles River Labs, Wilmington, MA) and allowed to acclimate to the animal facilities at the Dallas VA Medical Center for one week. 5–1×10^7^ cells were suspended in 250 µl of growth media and then mixed with 250 µl (total volume of 500 µl) of Matrigel (BD Biosciences, Franklin Lakes, NJ) and implanted under the skin of the mouse in the dorsal flank; SEG-1 lung adenocarcinoma cells or OE33 esophageal adenocarcinoma cells were used as a positive control. Mice were assessed daily for tumor formation and growth. At sacrifice, tumors were removed and processed by fixing in 4% formaldehyde solution followed by immersion in 10% neutral buffered formalin, dehydration, and paraffin embedding. Five micron paraffin-embedded sections were stained with hematoxylin and eosin (H&E) for histologic assessment. Calipers were used to measure the tumor length and width. The volume of each tumor was determined using the modified ellipsoidal formula Volume =  ½ (length X width^2^); the resulting tumor volumes were then averaged.

### Statistical Analyses

Quantitative data are expressed as the mean + the standard error of the mean (SEM). Statistical analysis was performed using ANOVA and the Student-Newman-Keuls multiple-comparison test with the Instat for Windows statistical software package (GraphPad Software, San Diego, CA). P values <0.05 were considered significant for all analyses.
